# The role of extracellular vesicles in the context of (inter‐)cellular communication contributing to adipose tissue dysfunction in lipedema

**DOI:** 10.3389/fcell.2026.1804905

**Published:** 2026-03-31

**Authors:** Katharina Helena Morawitz, Julia Christina Gross

**Affiliations:** Institute of Molecular Medicine, Health and Medical University, Potsdam, Germany

**Keywords:** extracellular vesicles, fibrosis, gender gap, hormones, lipid droplets, obesity

## Abstract

Lipedema is a chronic, female-predominant disorder of subcutaneous adipose tissue characterized by disproportionate fat expansion, pain, and fibrosis. Despite its high prevalence, the cellular mechanisms underlying lipedema remain poorly understood. While the clinical features have been extensively described, its biology of adipose tissue dysfunction and aberrant intercellular communication is still unclear. In comparison to obesity, lipedema is marked by local dysregulation of adipocyte–stromal and adipocyte–vascular interactions. In this hypothesis perspective, we discuss emerging mechanistic concepts from a cell biology perspective that are particularly relevant to lipedema, focusing on (i) organelle contact site dynamics in adipocytes and their role in lipid handling and stress adaptation; (ii) extracellular vesicle (EV)–mediated crosstalk between endothelial cells, adipocytes, and immune cells as a driver of localized inflammation and fibrosis; and (iii) estrogen-linked signaling pathways that may imprint EV cargo and cellular behavior in a sex-specific manner. By integrating these perspectives, we highlight open experimental settings and mechanistic parallels to other adipose tissue pathologies that help understanding lipedema as a distinct cellular and molecular entity. Investigating how organelle biology, extracellular vesicles communication and hormonal context intersect in adipose tissue may uncover novel biomarkers and therapeutic entry points for this long-neglected condition.

## Introduction

Lipedema is a chronic adipose tissue disorder characterized by a symmetrical but disproportional accumulation of subcutaneous fat, predominantly affecting the lower extremities (Herbst, 2012; Wold et al., 1951). The condition occurs almost exclusively in women, and an endocrine contribution is likely as lipedema typically arises and progresses during hormonal change, such as puberty, pregnancy or menopause (Herbst, 2012; Kruppa et al., 2020). Lipedema affects up to 10%–15% of women and remains widely underdiagnosed, frequently being misclassified as obesity or lymphedema (Fife et al., 2010; Herbst et al., 2021). Clinical symptoms of lipedema include painful, pressure-sensitive adipose tissue, increased capillary fragility leading to easy bruising, and a sensation of heaviness in the affected limbs (Forner-Cordero et al., 2012). Disease progression is characterized by fibrotic remodeling of adipose tissue and secondary lymphatic alterations aggravating the symptoms (Al-Ghadban et al., 2019).

**FIGURE 1:**
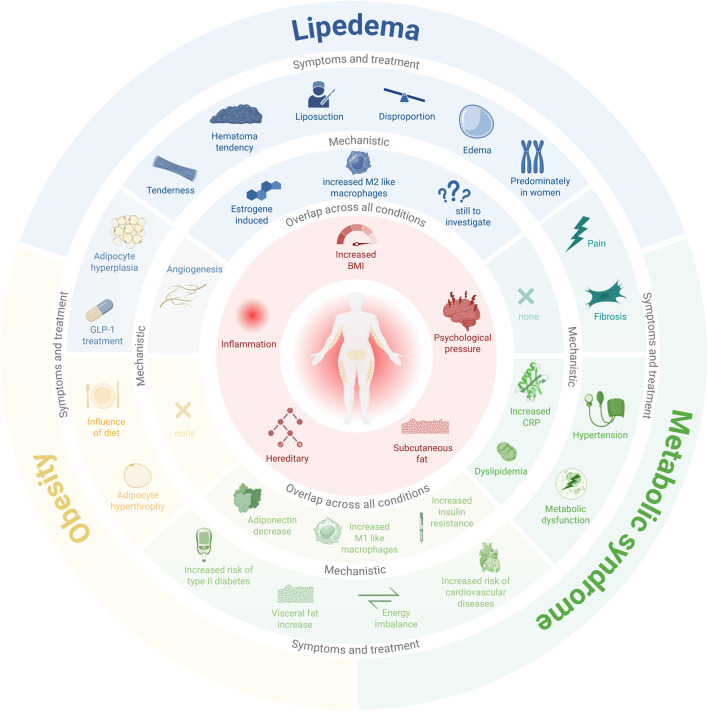
Overview of shared and disease-specific symptoms, treatments and mechanistic aspects across lipedema, obesity, and metabolic syndrome. The outermost ring labels the three conditions lipedema (blue), obesity (yellow), and metabolic syndrome (green). The inner rings are organized to depict symptoms and associated treatment as well as mechanistic aspects that are specific to a single condition or shared between two or all three conditions, as indicated by their spatial overlap across segments. Central elements indicate features common to all conditions, including inflammation, psychological pressure, hereditary factors, and increased BMI and increased subcutaneous fat. Despite the substantial overlap in symptoms among these diseases, there are distinctive differences that enable differentiation and subsequent diagnosis through further examination (Forner-Cordero et al., 2012; Grundy et al., 2004; Kahn et al., 2019; Kamamoto et al., 2024; Kruppa et al., 2020; Lustig et al., 2022; Ohashi et al., 2010; Poojari et al., 2022). Created in BioRender https://BioRender.com/933nmej.

Despite overlapping clinical features of fat accumulation and inflammation, lipedema is pathophysiologically distinct from both obesity and metabolic syndrome (Figure 1). In lipedema, fat accumulation is regionally restricted, largely independent of body mass index, and resistant to caloric restriction and exercise (Herbst, 2012). While metabolic syndrome is characterized by systemic insulin resistance, dyslipidemia, and chronic low-grade inflammation driven predominantly by visceral adipose tissue (Kahn et al., 2019), lipedema primarily affects subcutaneous fat and is not necessarily associated with metabolic dysfunction (Kruppa et al., 2023) (Figure 1). These observations point toward fundamental differences in adipose tissue regulation and inflammatory signaling rather than simple alterations in energy balance.

At the cellular level, lipedema adipose tissue expansion is primarily associated with adipocyte hyperplasia, although secondary hypertrophy may occur locally during disease progression. This contrasts with obesity, in which visceral, hypertrophic adipocytes exhibit enhanced basal and catecholamine-induced lipolysis, altered adrenergic receptor expression, and impaired insulin-mediated suppression of lipolysis (Item and Konrad, 2012).

Emerging evidence indicates that lipedema is driven by intracellular and intercellular alterations in adipose tissue biology. Multi-omics analyses have identified distinct transcriptomic, proteomic, and metabolic signatures that distinguish lipedema from obesity and implicate dysregulated adipocyte differentiation, extracellular matrix remodeling, and immune signaling as core disease features (Straub et al., 2025). In addition, transcriptomic and cellular profiling has revealed a disease-specific immune microenvironment dominated by M2-like macrophages that actively influence adipocyte differentiation and tissue remodeling (Wolf et al., 2022). Extracellular vesicles (EVs) have emerged as key mediators of intercellular communication within adipose tissue, enabling the transfer of proteins, lipids, and nucleic acids between endothelial cells, adipocytes, immune cells, and stromal populations (Crewe et al., 2018). Together, these findings highlight aberrant intra- and intercellular communication within the adipose tissue niche as a central driver of lipedema pathogenesis.

In this hypothesis perspective, we highlight current molecular mechanisms underlying other adipose tissue disorders in comparison to lipedema, focusing on disturbed interactions between adipocytes and surrounding stromal and vascular cells, fibrotic tissue remodeling, and new concepts of vesicle-mediated communication that link metabolic, inflammatory, and hormonal signals. We highlight open experimental questions and conceptual frameworks that may help define lipedema as a distinct cellular and molecular entity and identify new avenues for targeted therapeutic intervention.

## Organelle contact site dysfunction in adipocytes

Within adipocytes lipid storage and mobilization rely on coordinated interactions between the endoplasmic reticulum (ER), lipid droplets (LDs), and mitochondria (Bohnert and Schrul, 2024). In lipedema, mechanistic dysregulation may arise from impaired lipid droplet biology and remobilization of stored lipids, leading to adipocyte hyperplasia and progressively to hypertrophy. Importantly, this expansion does not appear to reflect a complete failure of adipocyte function but rather a qualitative alteration in intracellular lipid handling, suggesting that fundamental aspects of organelle communication may be altered in lipedema adipocytes.

Lipid droplets originate at the ER when neutral lipids, such as triacylglycerols, accumulate between the two leaflets of the ER membrane and phase-separate to form nascent lipid lenses that bud into the cytoplasm (Bohnert and Schrul, 2024). Mature lipid droplets are surrounded by a phospholipid monolayer and decorated with specific surface proteins that regulate lipid storage and mobilization (Majchrzak et al., 2024). Extensive work over the past decade has demonstrated that lipid droplets are dynamic organelles positioned near both the ER and mitochondria, with contact sites that respond to ER stress, oxidative stress, and cellular metabolic demands to regulate lipid filling and depletion (Freyre et al., 2019). In Neutral Lipid Storage Diseases (NLSD), rare autosomal recessive disorders caused by mutations in either adipose triglyceride lipase (ATGL) and α-β hydrolase domain 5 (ABHD5) proteins lead to accumulation of LD in different tissues and cell types with the consequence of myopathy or ichthyosis (Missaglia et al., 2019).

Adipose tissue is highly plastic, with low-level turnover and replacement of approximately 10% of adipocytes per year in humans. Its regional distribution is influenced by physiological, hormonal and pharmacological cues and contains interconvertible cell populations, such as white, beige and brown adipocytes (Sakers et al., 2022). Brown and beige adipocytes are important for thermoregulation in infants and adults, respectively. White adipocytes continuously switch between two opposing metabolic programs: nutrient storage during energy surplus and nutrient release during fasting or increased energy demand, such as exercise or cold exposure. During lipolysis, stored triacylglycerols are hydrolyzed and free fatty acids are released into the circulation. During the transition from beige to white adipocytes, cells lose uncoupling protein 1 (UCP1) expression and mitochondrial density and remodel their lipid droplets from a multilocular to a unilocular architecture, a process that depends on mitochondrial clearance rather than progenitor re-entry (Altshuler-Keylin et al., 2016; Roh et al., 2018).

Disruption of organelle contact sites is a recognized feature of adipocyte dysfunction in other diseases: In obesity, altered ER–mitochondria contact sites are associated with ER stress, mitochondrial dysfunction, and inflammatory signaling (Monteiro-Cardoso and Giordano, 2024). Conversely, in lipodystrophic conditions, defects in lipid droplet biogenesis or lipolysis lead to an inability to properly store or mobilize lipids (Duan and Savage, 2024). Lipolysis is tightly regulated by the lipid droplet surface protein perilipin 1 (PLIN1), which controls access of ATGL and hormone-sensitive lipase to stored lipids through phosphorylation-dependent interactions with CGI-58 and protein kinase A signaling (Granneman et al., 2009). Perilipins (PLIN1–PLIN5) are a family of lipid droplet–associated proteins that differentially associate with distinct LD populations within cells, reflecting varied roles in lipid storage and mobilization across tissues and metabolic states (Chouchani and Kajimura, 2019; Sztalryd and Brasaemle, 2017). Similarly, in *Drosophila* fat body it was shown that peripheral LDs in contact with the plasma membrane change during fasting and high nutrient diet, while distinct, larger LDs promote long-term triacylglycerol storage (Ugrankar et al., 2019). In addition to classical lipolysis, lipids can also be exported from adipocytes via small EVs, providing local signals that influence macrophage differentiation and tissue remodeling (Flaherty et al., 2019).

These observations raise the possibility that endoplasmic reticulum–lipid droplet–mitochondria communication may be altered in lipedema adipocytes, such that it would allow progressive lipid accumulation without efficient oxidation, remodeling, or mobilization, while delaying overt metabolic failure.

## Vascular and inflammatory features of lipedema adipose tissue

Histological analyses of lipedema adipose tissue consistently report fragile and dilated capillaries, increased vascular permeability, and signs of microangiopathy (Kruppa et al., 2020). These alterations are likely to influence local inflammation and adipocyte behavior. Fatty acid release from dying adipocytes may elicit inflammatory responses, although the impact varies across studies (Tilg et al., 2020). Adipose-tissue residing macrophages clear dying adipocytes and recruit adipocyte progenitor cells, linking inflammation with adipogenesis (Lee et al., 2013). In contrast, adipocyte-derived adiponectin promotes an anti-inflammatory phenotype in macrophages, highlighting the importance of balanced adipokine signaling for tissue homeostasis (Ohashi et al., 2010). Beyond adipocytes and immune cells, stromal populations actively shape the inflammatory milieu. Notably, fibro-inflammatory progenitors have been identified as a distinct fibroblast subset that promotes macrophage inflammation during adipose tissue expansion, underscoring the complexity of cellular interactions within dysfunctional fat depots (Hepler et al., 2018). Microvascular dysfunction may further exacerbate these processes through local hypoxia. Hypoxic conditions are thought to arise from adipocyte hypertrophy, which can exceed the diffusion limit of oxygen, as well as from reduced vascular density within expanding adipose tissue (Trayhurn, 2013). In obesity, hypoxia-inducible factor 1α (HIF1α) drives pro-inflammatory and pro-fibrotic gene expression activation, contributing to extracellular matrix deposition and tissue stiffening (Halberg et al., 2009; Lee et al., 2014). Similar hypoxia-driven mechanisms may operate in lipedema, linking microvascular alterations to inflammation and fibrosis.

## The Wnt–EV axis: linking vascular dysfunction, fibrosis, and adipocyte fate

**FIGURE 2:**
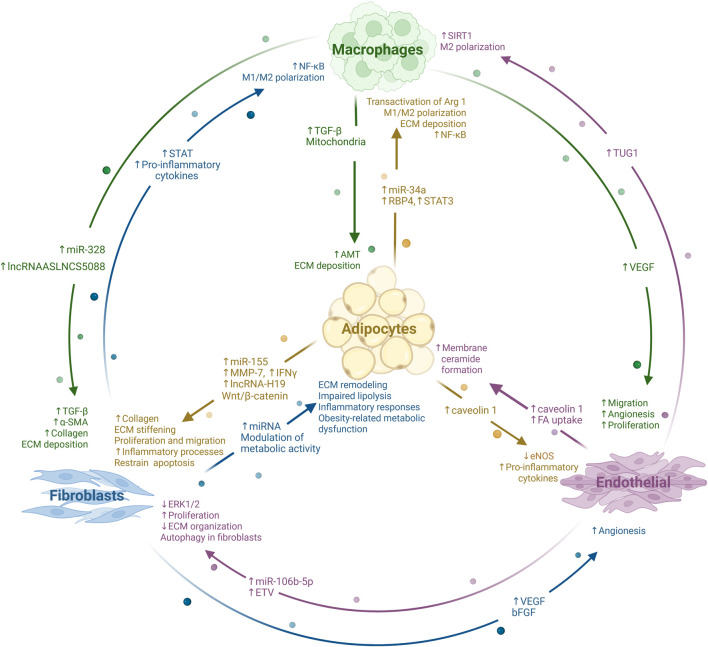
Intercellular communication via extracellular vesicles in adipose tissue. Fibroblasts, macrophages, endothelial cells and adipocytes exchange extracellular vesicles (EVs) that carry cytokines, microRNAs, long non-coding RNAs, and other signaling molecules, thereby shaping local inflammation, fibrosis, and adipose tissue remodeling. Fibroblast- and macrophage-derived EVs can promote macrophage polarization and activate pro-inflammatory and pro-fibrotic signaling pathways, including NF-κB and TGF-β signaling (Hua et al., 2024) resulting in the deposition and stiffening of the extracellular matrix (ECM) (Wang et al., 2020; Zhang et al., 2024). Fibroblast EV induce metabolic dysfunction and inflammatory responses in adipocytes, which react with elevated levels of miR-155 and MMP-7, as well as IFNγ, (Martín-Taboada et al., 2022). Through the upregulation of VEGF and bFGF of fibroblasts and macrophages EVs, the angionesis as well as the proliferation and migration in endothelial cells is upregulated (Xue et al., 2025; Yuan et al., 2025). As a response endothelial cells release EVs with upregulated TUG1 which leads to an upregulation of SIRT1 and ultimately in M2 polarization in macrophages. Endothelial EVs taken up from Fibroblasts contain upregulated ETV and miR-106b-5p which leads in fibroblasts to autophagy, downregulation of ERK1/2 and ECM organization as well as upregulation in proliferation (Fang et al., 2024; Ma et al., 2021; Yuan et al., 2025). Adipocyte-derived EVs can promote proliferation and migration of fibroblasts via lncRNA-H19 (Wang et al., 2020) or play a role in the polarization of M1 and M2-like macrophages (Le Lay et al., 2021; Tang et al., 2022). Endothelial-cells and adipocytes exchange caveolin 1 protein through EVs resulting in upregulation od pro-inflammatory cytokines and downregulating of the eNOS in endothelial cells and in an upregulation of membrane ceramide formation in adipocytes (Crewe et al., 2018; Peche et al., 2023). Created in BioRender https://BioRender.com/k632ciz.

Wnt signaling plays a central role in adipose tissue biology and mechanistically links vascular dysfunction, altered adipocyte differentiation and fibrosis (Chen and Wang, 2018). Canonical Wnt/β-catenin signaling suppresses adipogenesis by inhibiting the transcription factors peroxisomal proliferator-activated receptor (PPARγ) and CCAAT enhancer binding protein (C/EBPα), thereby maintaining progenitor cells in an undifferentiated state (Ross et al., 2000). Although traditionally associated with early adipogenesis, Wnt/β-catenin signaling persists in a subset of mature adipocytes and continues to influence adipocyte function beyond differentiation (Liu et al., 2022). In parallel, non-canonical Wnt ligands, particularly Wnt5a, are upregulated in hypertrophic adipose tissue via YAP signaling and promote pro-inflammatory remodeling, fibrosis, and vascular dysfunction (Lee et al., 2022). Importantly, Wnt ligands are secreted not only as soluble factors but also via EVs (Gross et al., 2012). Adipocyte-derived EVs carry bioactive proteins, lipids, and nucleic acids that regulate adipocyte homeostasis, endothelial behavior and immune activity (Zhou et al., 2020). Alterations in EV cargo composition have been demonstrated in obesity and metabolic disease (Malaguarnera et al., 2025), and recent work demonstrates that EV cargo reflects the lipid composition of the parental adipocyte, enabling lipid imbalances to directly shape intercellular signaling (Le Lay et al., 2021). Experimental evidence from other tissues supports a close coupling between Wnt signaling, EV biology, and fibrotic remodeling. Sustained Wnt activation in dermal progenitor cells induces fibrotic extracellular matrix thickening and lipodystrophy of dermal white adipose tissue, phenotypes that are reversible upon withdrawal of Wnt activation (Ma et al., 2020). Conversely, inhibition of Wnt signaling through the secreted Wnt antagonist NOTUM reduces fibrosis and inflammation in adipose tissue, promotes thermogenic differentiation, and improves metabolic homeostasis (Guo et al., 2021). Aberrant Wnt activation has also been linked to EV-related proteostasis pathways in failing cardiomyocytes, highlighting the general relevance of Wnt–EV interactions in fibrotic remodeling and disease progression (Schoger et al., 2023) (Figure 2).

## EVs as mediators of vascular–adipocyte communication

Endothelial-derived EVs influence adipocyte differentiation and their metabolic state, and *vice versa* adipocyte-derived EVs modulate endothelial function and immune cell activation, suggesting a multidirectional signaling loop that can reinforce local tissue states. Cell-fate decisions of adipocyte progenitor cells (APCs) are particularly sensitive to such local cues. Alterations in the EV milieu or vascular signaling environment may bias these progenitors toward maladaptive, pro-fibrogenic differentiation pathways, thereby contributing to fibrosis and impaired adipose tissue plasticity (Gan et al., 2025; Higos et al., 2025). Lipedema adipocytes display a senescence-associated secretory phenotype (SASP) resulting in the continuous release of pro-inflammatory cytokines (e.g., IL-11, IL-28A, IL-29) and ECM components, which can promote local fibrosis and immune cell recruitment (Ishaq et al., 2022; Wolf et al., 2022). In related adipose tissue and fibrotic contexts, SASP factors are packaged into adipose-derived extracellular vesicles (ADEVs), where they amplify inflammatory signaling and tissue remodeling (Huang and Xu, 2021; Li et al., 2020). Such SASP-EVs can propagate the senescent phenotype to neighbouring stromal-vascular cells and mesenchymal stromal cells, including altered phosphatase and tensin homolog (PTEN) nuclear import and insulin signalling, thereby reinforcing a chronic inflammatory loop (Chechekhina et al., 2025). The EV cargo (comprising cytokines, microRNAs and proteins) has also been demonstrated to promote macrophage infiltration and angiogenic changes, thus may contribute to the characteristic pain and easy bruising of lipedema (AL-Ghadban et al., 2019; Poojari et al., 2022). ADEVs convey fibrotic signals locally rather than systemically, and may thus help to explain the persistence of pathological fat accumulation in lipedema despite conventional weight-loss interventions (Kamamoto et al., 2024; Kruppa et al., 2020).

Recent studies have demonstrated discrepancies in the profile of microRNA carried through ADEVs between individuals with lipedema and those who are healthy. Specifically, the analysis of microRNAs (miRNAs) 155, 328, and 34a revealed their distinct expression patterns in EVs isolated from these two groups. This observation suggests that the communication mechanism between cells is altered in individuals with lipedema, potentially leading to the development and progression of this condition (Cione et al., 2024; Priglinger et al., 2020).

Collectively, these findings suggest that dysregulation of Wnt signaling and EV-mediated communication may be relevant to altered adipocyte differentiation and hyperplasia, vascular function and fibrotic remodeling in lipedema.

## Estrogen-linked EV signaling

Lipedema commonly manifests or worsens during specific hormonal transitions throughout lifespan, such as puberty, periods of pregnancy, and menopause (Ahmed et al., 2022; Katzer et al., 2021; Ko and Jung, 2021), suggesting that estrogen signaling plays a pivotal role in the pathogenesis of the disease. In rare male cases, lipedema has been associated with lower testosterone and/or relatively higher estrogen levels compared with typical male hormone profiles (Hess and Cooke, 2018; Katzer et al., 2021). Estrogen, as the key female reproductive hormone binds to different estrogen receptors (ER α and β) to regulate gene transcription and has significant impacts on various body systems, including the endocrine, cardiovascular, metabolic, bone growth and maturation, and skin.

In lipedema the balance of estrogen receptors in lowerbody subcutaneous adipocytes is shifted toward a higher ER α to ER β ratio. This change reduces the suppressive influence of ER β on ER α driven gene programs and creates a cascade that favors fat accumulation. Higher ER α dominant signaling in subcutaneous fat promotes lipid uptake and storage, while relatively higher ER β expression in visceral fat limits adipogenesis, accounting for the distinct estrogen driven fat distribution observed (Katzer et al., 2021). Enhanced ER β activity has been linked to altered mitochondrial function and suppression of adipogenic programs, resulting in lipodystrophy-like phenotypes in experimental models (Foryst-Ludwig et al., 2008; Ventura-Clapier et al., 2019), suggesting a role for sex-hormone–dependent regulation of adipocyte organelle function in lipedema. Hormonal signaling has been shown to regulate key steps of EV biogenesis through pathways such as PI3K/Akt and Hippo-YAP. In turn, EV-associated molecules, including microRNAs and RNA-binding proteins such as YBX1, can modulate hormone-responsive signaling pathways (Bi et al., 2025). Experimental studies in hormone-responsive systems have demonstrated that estrogen alters EV release in a dose-dependent manner and influences EV cargo composition, supporting the concept of reciprocal regulation between endocrine signaling and EV-mediated communication. For example, 17β-estradiol has been reported to increase EV release and modulate EV microRNA cargo, including enrichment of let-7 family members, with differences linked to menopausal status (Drula et al., 2023).

Fibrotic and inflammatory pathways are relevant for lipedema and obesity-related adipose tissue dysfunction. In lipedema, adipose tissue is characterized by an increased abundance of anti-inflammatory M2-like macrophages expressing the scavenger receptor CD163, a feature that distinguishes it from obesity-associated adipose inflammation (Grewal et al., 2025). While blockade of CD163 has been proposed as a potential therapeutic approach, its functional relevance in lipedema requires further investigation. In contrast, obesity and estrogen deficiency are associated with chronic low-grade inflammation (“metainflammation”), driven in part by pro-inflammatory M1 macrophages producing cytokines such as TNF and IL-1β (Marsh et al., 2023; Zhou et al., 2020). Consistent with a protective role of estrogen signaling, deletion of ERα in adipocytes leads to increased adipose tissue inflammation and fibrosis in animal models (Zhou et al., 2020). Evidence for fibrotic remodeling in lipedema includes transcriptomic upregulation of extracellular matrix components such as lumican (Grewal et al., 2025). Paracrine signaling further contributes to immune modulation within adipose tissue. EVs derived from estrogen-stimulated cells have been shown to influence macrophage activation by transferring microRNAs that suppress inflammatory markers, including IFN-γ and IL-12A, in recipient macrophages (Drula et al., 2023) (Figure 2).

## Conclusion

Although clinical features of lipedema are increasingly well described, the cellular and molecular mechanisms remain to be fully understood. The evidence summarized here show how lipedema differs from obesity and metabolic syndrome with respect to fat distribution, immune composition, and metabolic regulation. Alterations in adipocyte expansion, microvascular structure, and macrophage polarization point toward a locally dysregulated adipose tissue niche that is not primarily driven by systemic metabolic dysfunction. Growing evidence suggests that extracellular vesicle–mediated crosstalk and hormonal context contribute to localized inflammation and fibrotic remodeling, although their precise roles in adipose tissue remain to be defined. How hormone-regulated EV signaling, Wnt pathway activity, and organelle communication directly contribute to lipedema pathophysiology remains to be established. Future studies integrating patient-derived tissue analyses with functional and longitudinal approaches will be essential to clarify how these pathways intersect in lipedema. A better understanding of adipocyte–vascular–immune crosstalk and EV-mediated signaling may help refine disease classification and identify biomarkers or therapeutic strategies tailored to this underrecognized condition.
